# Laparoscopic Repair of an Acute Traumatic Diaphragmatic Hernia: Clinical Case

**DOI:** 10.7759/cureus.11082

**Published:** 2020-10-21

**Authors:** Filipa Campos Costa, Vasco Cardoso, Ana Maria Monteiro, José Guerreiro

**Affiliations:** 1 Surgery, Hospital de São Francisco Xavier, Lisbon, PRT

**Keywords:** trauma, emergency surgery, laparoscopy, traumatic diaphragmatic hernia, diaphragmatic injury

## Abstract

Traumatic diaphragmatic rupture is uncommon, life threatening and remains a diagnostic and radiographic challenge. Diagnosis is frequently delayed, which may result in a late intervention with a potential catastrophic outcome. We report a case of an acute diaphragmatic laceration in a 40-year-old woman, with a personal history of bipolar disease, admitted on the emergency department after falling from a nine-meter building. During initial evaluation, the plain chest radiograph showed multiple rib fractures associated with a significant left pneumothorax. It also showed an elevated left diaphragm with a suspicious gastric shadow in the left hemithorax. Computed tomography confirmed the diagnosis of a left-sided diaphragmatic laceration and the patient was advised surgical intervention. During laparoscopy, a 7 cm rupture of the left hemi-diaphragm with herniation of the stomach was identified. The hernia was reduced laparoscopically and the defect repaired with interrupted, non-absorbable, sutures. The patient had an uneventful recovery and remained well at a 3-month follow-up visit. Emergency repair of the diaphragm is usually performed via a thoracotomy or/and laparotomy. However, if the patient is hemodynamically stable and major organ injuries have been excluded, a laparoscopic approach can be considered safe and effective.

## Introduction

Diaphragmatic rupture is an infrequent and life-threatening complication of trauma. As the signs and symptoms of acute diaphragmatic trauma are often masked by severe concomitant injuries to other organs, a high índex of suspicion is warranted for clinical diagnosis, especially in the absence of pathognomonic radiographic findings [1,2]. Diagnosis is usually delayed and patients may be asymptomatic for years after trauma, until complications occur [3]. Once diagnosed, left-sided diaphragmatic injuries should be repaired to reduce the risk of subsequent complications [4]. Laparotomy or thoracotomy are the traditional treatment approaches, the choice being largely dependent on the skill set of the surgeon involved [2,3,4]. The advent of laparoscopy has provided a new approach to this clinical situation. However, reports of laparoscopic or laparoscopic-assisted diaphragmatic hernia repair are scarce and are generally limited to chronic post-traumatic or congenital hernias [4].

## Case presentation

A 40 year-old woman was admitted to the emergency department after falling from a nine-meter building. The patient complained of left hemithorax pain and shortness of breath, left leg and lower back pain.

**Figure 1:**
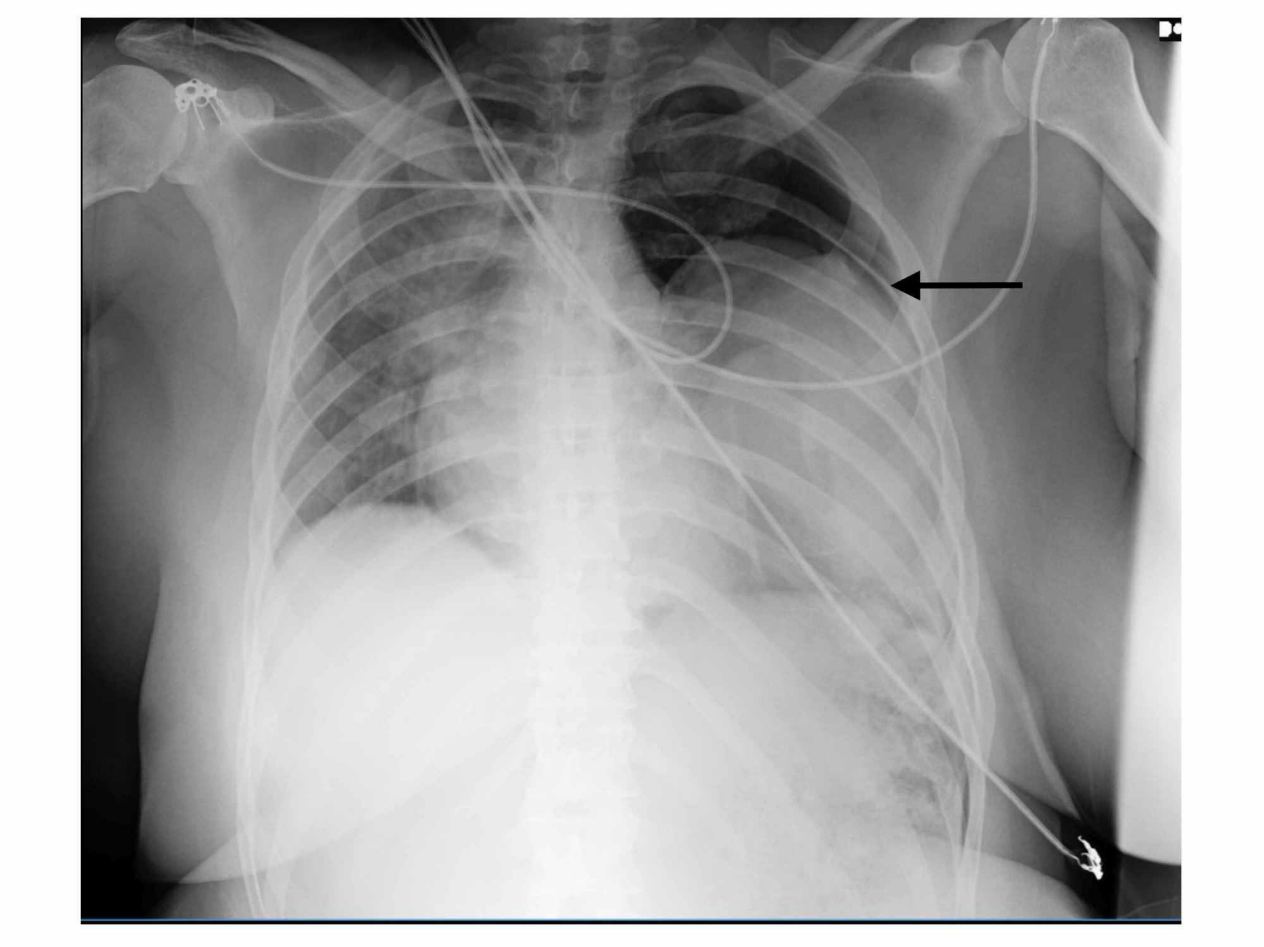
X-ray showing a left pneumothorax and a suspicious gastric shadow in left hemi-thorax

On initial evaluation, her vital signs were stable. Chest evaluation revealed respiratory distress on 6L/min oxygen support, pain on palpation and loss of breath sounds on the left hemithorax. The abdomen was mildly tender to deep palpation and she could not move her left leg due to pain. Aside from having a bipolar disorder, followed by regular psychiatry appointments, she lacked a significant past medical history.The patient was evaluated and stabilized in the emergency room. Chest radiograph showed multiple rib fractures and a left pneumothorax. It also showed an elevated left diaphragm with suspicious gas pattern of the stomach (Figure 1).

The chest radiograph was compared to past x-rays taken during a previous hospital admission and none of the described alterations were present before.

**Figure 2:**
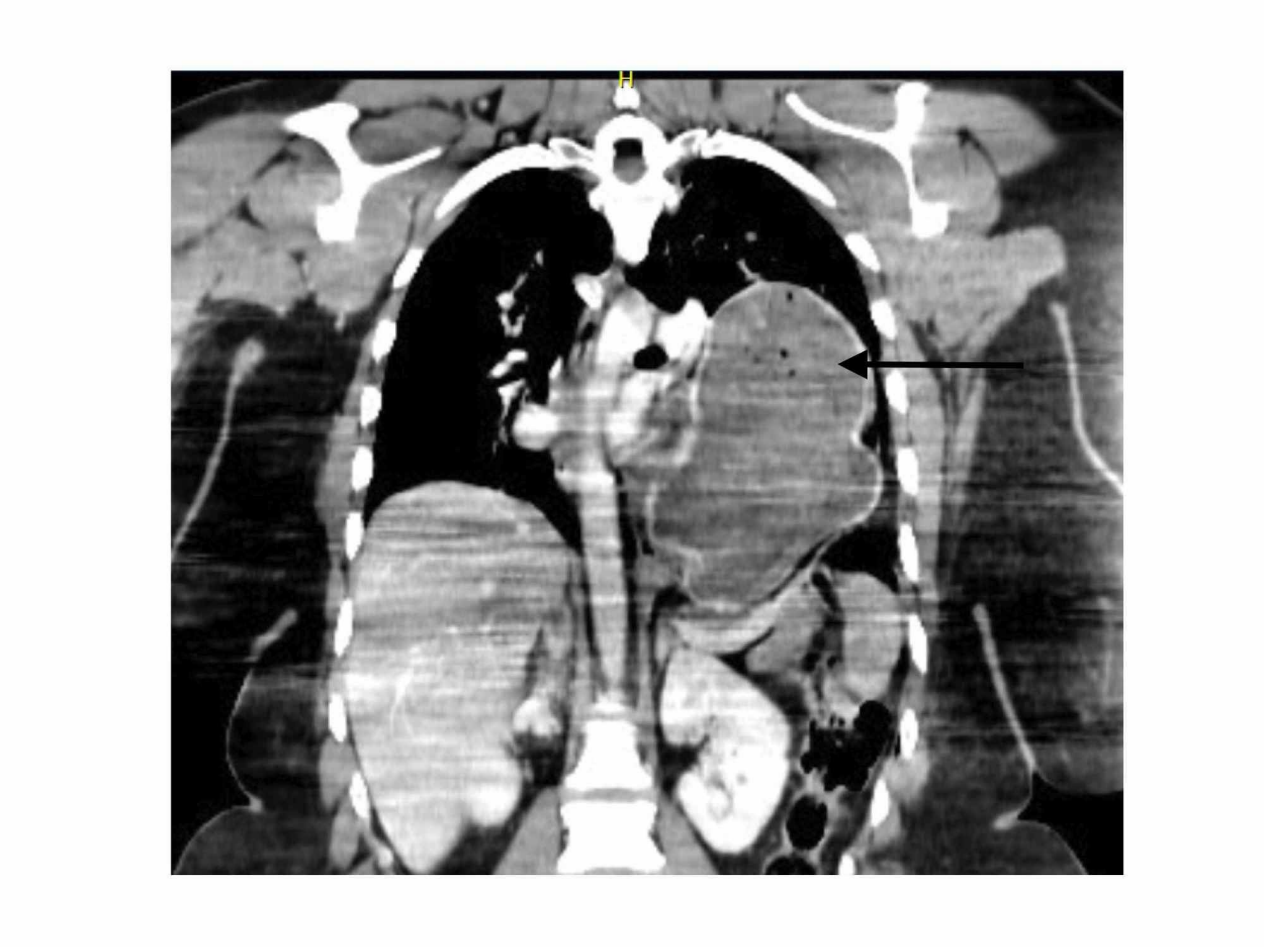
CT section showing herniation of stomach in left hemi-thorax

**Figure 3:**
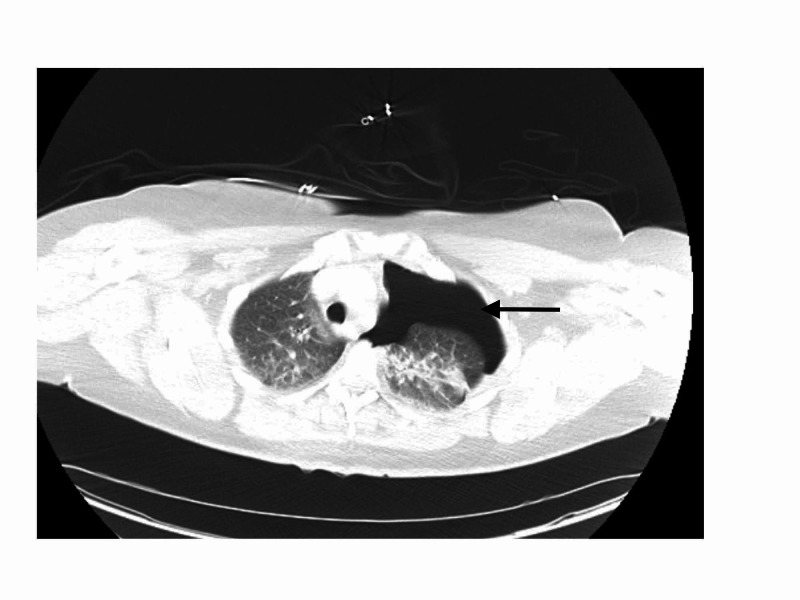
CT scan showing a left pneumothorax

Computed tomography (CT) scan of the thorax and abdomen revealed the presence of a left pneumothorax with associated costal fractures. It also showed an acute rupture of the left hemi-diaphragm causing herniation of the stomach and subsequent lung atelectasis (Figure 2, 3).

No other thoracic or abdominal injuries were reported. Other minor associated injuries such as left tibia fracture were identified. The patient was advised for a minimally invasive surgical intervention and taken to the operating room.

**Figure 4:**
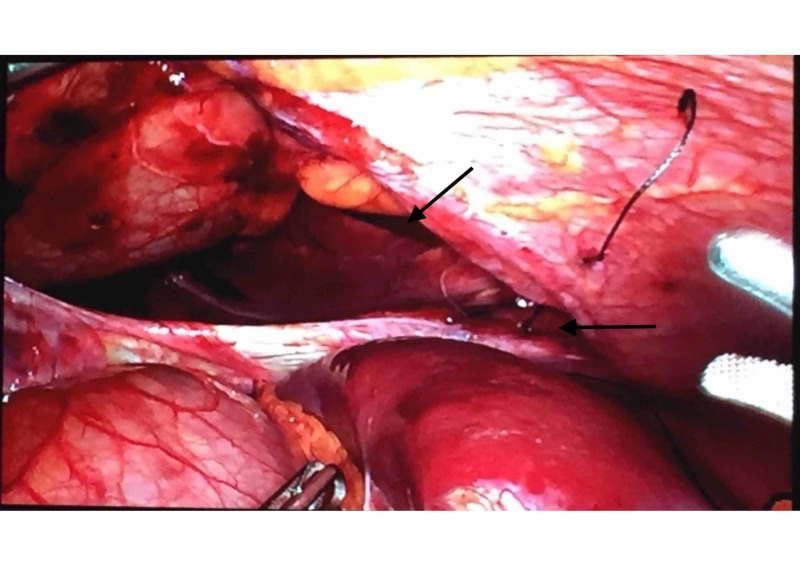
Laparoscopic image showing the diaphragmatic defect

After placing a left chest tube in the second intercostal space, an exploratory laparoscopy was performed. The greater omentum and the stomach were found within the left hemithorax, protruding through a seven-centimeter diaphragmatic defect (Figure.4).

**Figure 5:**
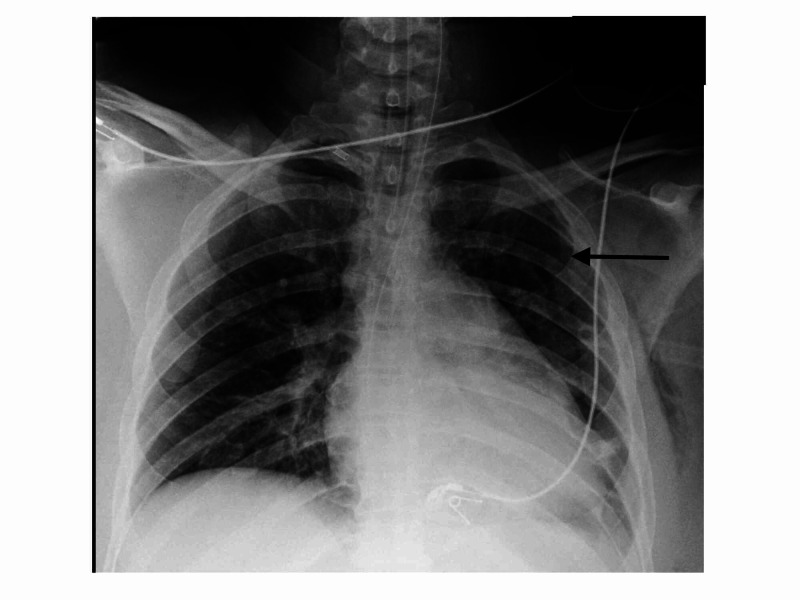
Post-operative x-ray

After reducing the hernia contents, a tension-free repair was performed with interrupted silk suture; the use of an absorbable mesh was considered unnecessary. No other signs of injury were identified upon final inspection of the abdominal cavity. The patient’s postoperative course was uneventful with complete expansion of the right lung (Figure 5).

The patient was discharged on the fifth postoperative day after psychiatric and orthopedic evaluation. On the third month follow-up, the patient was clinically asymptomatic and no evidence of recurrence was identified on the x-ray.

## Discussion

Diaphragmatic hernia is a herniation of the abdominal organs or tissues into the thoracic cavity. Traumatic diaphragmatic injuries are rare and usually occur after thoracic or abdominal blunt (2.9%) or penetrating (3.4%) trauma [4,5,6].

However, the precise incidence of this injury is likely to be under-reported. Diaphragmatic injuries, in the absence of acute diaphragmatic hernias, are often missed by diagnostic imaging [1]. The diagnosis can be difficult to make as the physical examination may be unremarkable, and imaging may initially fail to reveal the injury [1].

The possibility of a diaphragmatic injury should always be considered in the context of rapid deceleration or crush injuries [1]. There are a lot of hypotheses about the mechanism of delayed presentation of a diaphragmatic rupture. Delayed rupture of a devitalised diaphragmatic muscle may occur several days or weeks after the initial injury [6].

Most of the traumatic diaphragmatic injuries (80-90%) occur in the left diaphragm because the left diaphragm is congenitally weaker than the right diaphragm which is protected by the liver [6]. The clinical presentation is varied as patients may be asymptomatic, may have an acute presentation as shortness of breath, shoulder pain, epigastric pain or vomiting, or may manifest at a later stage, after adhesions are formed, as intestinal obstruction, strangulation or perforation [2,7].

The symptoms and signs are strongly associated with the size of the diaphragmatic defect, herniated organs and the existence of pulmonary disease [6]. During initial assessment for trauma, most casualties will have a chest radiograph taken. The chest radiograph is an integral adjunct in the Advanced Trauma Life Support guidelines for the initial evaluation of the trauma patient, and is often the first clue to the presence of an acute diaphragmatic injury [2,4].

However, a chest radiograph may not be useful in some cases as signs are often masked by associated lung contusion, hemothorax, pneumothorax, pleural effusion, atelectasis, emphysema and non-specific elevation of diaphragm [7]. Between 20-50% of patients who are later found to have a traumatic diaphragmatic injury have their initial trauma chest radiographs described as normal [4].

CT scan of the chest has become an essential tool for the evaluation of a hemodynamically stable trauma patient. In the absence of an acute hernia, CT scans offer little benefit compared with conventional plain radiographs, as the sensitivity of the CT for the diagnosis of isolated diaphragmatic injury is limited. However, in the presence of herniation of abdominal organs into the thoracic cavity, the sensitivity of oral contrast-enhanced CT scan is close to 95%. It is especially helpful if the plain chest radiograph is obscured by the presence of a hemothorax or a lung contusion [1,7].

Traumatic rupture of the diaphragm is considered an indication for surgical repair, especially in symptomatic patients. The onset of complications carries highest mortality and morbidity rates. This makes emergency surgery mandatory [3].

Two principles must be followed when repairing acute traumatic diaphragmatic hernias: complete reduction of the herniated organs back into the abdomen and watertight closure of the diaphragm to avoid recurrence. Repair with non-absorbable simple sutures is adequate in most cases and the use of a mesh should be reserved for chronic and large defects. In some series, suture with absorbable sutures are associated with a higher rate of recurrence [4].

In patients with giant diaphragmatic hernias, synthetic or biologic grafts can be used for a tension free repair and prevention of recurrence [6]. It should always be considered that, the use of prosthetics may be of benefit in the repair of chronic diaphragmatic injury, but it carries a high rate of infection in the acute setting, especially in the presence of hollow viscous injury in the abdomen [4].

Laparotomy or thoracotomy are the traditional surgical approaches for patients with diaphragmatic rupture, the choice being largely dependent on the skill set of the surgeon involved [3,4].

However, given the high rate of associated injuries to intra-abdominal organs, it is generally recommended to approach the diaphragmatic injury through an abdominal approach rather than thoracic [1]. Laparoscopic approach is preferred in hemodynamically stable patients as it offers the benefits of less postoperative pain, faster recovery and a decreased risk of wound complications. Open surgical procedures are associated with increased postoperative pain, increased hospital length of stay, and development of long-term complications, such as an incisional hernia [2].

For these reasons, laparoscopic approaches for repair of hernias have gained in popularity being an excellent diagnostic and therapeutic approach in the setting of suspected diaphragmatic rupture [3,5]. Outcomes of acute diaphragmatic hernia repair are largely dictated by the severity of concomitant injuries. Reported mortality rates vary between 18% and 40% depending on whether the mechanism of trauma is blunt or penetrating and if associated with other injuries [4].

## Conclusions

Diaphragmatic injury is a rare entity and carries a significant morbidity and mortality, usually from associated injuries and delayed diagnosis. There are no specific signs and symptoms for diagnosing diaphragmatic rupture, the key being a high index of suspicion.

Reports of laparoscopic repair of traumatic diaphragmatic rupture are uncommon. However, in hemodynamically stable patients, laparoscopy should be considered as a diagnostic and therapeutic approach. It is safe and effective in repairing the diaphragm of selected patients following blunt abdominal trauma.
